# Functional connectivity and structural analysis of trial spinal cord stimulation responders in failed back surgery syndrome

**DOI:** 10.1371/journal.pone.0228306

**Published:** 2020-02-19

**Authors:** Peter A. Pahapill, Guangyu Chen, Elsa V. Arocho-Quinones, Andrew S. Nencka, Shi-Jiang Li

**Affiliations:** 1 Department of Neurosurgery, U.S. Department of Veterans Affairs Medical Center, Milwaukee, Wisconsin, United States of America; 2 Department of Neurosurgery, Medical College of Wisconsin, Milwaukee, Wisconsin, United States of America; 3 Department of Biophysics, Medical College of Wisconsin, Milwaukee, Wisconsin, United States of America; 4 Center for Imaging Research, Medical College of Wisconsin, Milwaukee, Wisconsin, United States of America; University at Buffalo, UNITED STATES

## Abstract

**Background:**

Chronic pain has been associated with alterations in brain structure and function that appear dependent on pain phenotype. Functional connectivity (FC) data on chronic back pain (CBP) is limited and based on heterogeneous pain populations. We hypothesize that failed back surgery syndrome (FBSS) patients being considered for spinal cord stimulation (SCS) therapy have altered resting state (RS) FC cross-network patterns that 1) specifically involve emotion and reward/aversion functions and 2) are related to pain scores.

**Methods:**

RS functional MRI (fMRI) scans were obtained for 10 FBSS patients who are being considered for but who have not yet undergone implantation of a permanent SCS device and 12 healthy age-matched controls. Seven RS networks were analyzed including the striatum (STM). The Wilcoxon signed-rank test evaluated differences in cross-network FC strength (FCS). Differences in periaqueductal grey (PAG) FC were assessed with seed-based analysis.

**Results:**

Cross-network FCS was decreased (p<0.05) between the STM and all other networks in these FBSS patients. There was a negative linear relationship (R^2^ = 0.76, p<0.0022) between STM_FCS_ index and pain scores. The PAG showed decreased FC with network elements and amygdala but increased FC with the sensorimotor cortex and cingulate gyrus.

**Conclusions:**

Decreased FC between STM and other RS networks in FBSS has not been previously reported. This STM_FCS_ index may represent a more objective measure of chronic pain specific to FBSS which may help guide patient selection for SCS and subsequent management.

## Introduction

Increasing evidence suggests a critical role of central nervous system (CNS) plasticity in the development and maintenance of chronic low back pain (cLBP). Understanding altered neural networks implicated in the pathophysiology of cLBP, in turn can lead to development of new management strategies. Resting-state (RS) functional connectivity magnetic resonance imaging (fcMRI) is a powerful tool for elucidating the areas of the brain involved in cLBP perception and modulation,[1–3] as it facilitates study of both local and diffuse functional properties in the undisturbed state of chronic pain and because RS networks (RSNs) are an intrinsic property of the brain found across various behavioral and physiological states.[1, 4] Human brain imaging studies have identified potential anatomical and functional biomarkers that differentiate cLBP patients from healthy subjects.[5] Chronification of LBP has been associated with alterations in brain anatomy and function, including a shift in activity from brain regions involved in acute pain to more emotion or reward circuitry.[1–3, 5] The reported changes can depend upon 1) the population of chronic pain patients being studied [1, 6] and 2) the duration of pain that the patients have endured.[1, 5, 7] Some of these structural and functional changes that occur with chronification of pain may be reversible with successful treatment.[8–11]

The belief that brain activity associated with chronic pain is localized to a discrete neural substrate (“pain matrix”) is no longer justifiable.[4] Instead, fcMRI studies show that multiple chronic pain conditions are associated with alterations in multiple intrinsic brain networks associated with sensory, motor, autonomic, cognitive, and emotional functions. Kucyi and Davis (2015) have championed the concept that chronic pain should be considered as a process encoded by a “pain connectome”, the spatiotemporal signature of brain network communication that represents the integration of all cognitive, affective and sensorimotor aspects of chronic pain.[4] This approach emphasizes the role of dynamic communication within and between networks in shaping cognition and pain behavior.[4, 12] Previous fcMRI studies on chronic pain patients have focused on changes within networks (Table 1). If one considers pain as a phenotypical conscious state, then analyzing functional connectivity changes between multiple interacting networks rather than relying on changes within individual cortical and subcortical networks may be more appropriate. As such, our goal is to consider a more integrative, whole-brain approach and study the relationships between several different RSNs (cross-network analysis), an approach that has been reported only once in pain patients.[12]

**Table 1:** Referenced studies with functional connectivity correlations with intensity and/or duration of chronic pain.

Reference	Study Details	Chronic Pain Group	Control Group	Main findings	Significance/Remarks
(1) Baliki MN, et al. 2014	RS fMRI Independent component network analysis; ROIs included: mPFC, Pre-Cu, ACC, LP, IFG, SMG, INS	18 CBP (5 F, 13 M) 19 CRPS (16 F, 3M) 14 OA (6 F, 8 M)	32 (24 F, 12 M)	CBP and CRPS patients show decreased MPFC and increased PreCu representation within the DMN. MPFC-INS connectivity showed high correlation to pain intesity in CBP (R = 0.75, p<0.01), CRPS (R = 0.71, p<0.01) and OA (R = 0.61, p<0.05) DMN high frequency spectral power shows significant positive correlation to pain duration in CBP (R = 0.65, p,0.01) and OA (R = 0.77, p,0.01), but not in CRPS (R = 0.11, p = 0.87)	MPFC exhibits connectivity changes in proportion to intensity of pain. The extent of association of the medial prefrontal component of the DMN with the insula, and its dissociation from the posterior components of the DMN, appears to be a function of the intensity of the chronic pain and the duration of its persistence
(3) Baliki MN, et al. 2012	Resting state (RS) fMRI Functional ROI for NAc, mPFC, INS determined from VBM	39 SBP (20 F, 19 M)	17 (7 F, 10 M)	Increased connectivity of nucleus accumbens with PFC predicted pain persistence. At one–year follow–up, there was decreased negative functional connectivity in SBPp between insula and dorsolateral prefrontal cortex (dLPFC) and precuneus (PreCu). This reduced functional connectivity was related positively with insula gray matter density and negatively with pain intensity.	Implication that corticostriatal circuitry may be involved in transition from acute to chronic pain. Implication that the functional reorganization of the insula may be coupled with gray matter changes and directly relate to the persistence of pain. Medications were controlled and results demonstrated medication-independent FC changes
(7) Yu R, et al. 2014	RS fMRI Seed-based analysis using vlPAG; ROIs: vmPFC/ACC, anterior insula, posterior insula, and amygdala	18 cLBP(12 F, 6 M)	18 (12 F, 6 M)	FC between the PAG and the ventral medial prefrontal cortex (vmPFC)/rostral anterior cingulate cortex (rACC) increased in cLBP patients compared to matched controls Negative correlations between pain ratings and PAG–vmPFC/rostral ACC FC in cLBP patients after pain-inducing maneuver. cLBP duration was negatively correlated with PAG–posterior insula and PAG–amygdala FC before any pain-inducing maneuver.	Did not find a correlation FC and severity of cLBP for ROIs at rest but did find a negative correlation between PAG-vmPFC/rostral ACC after pain-inducing maneurver (mechanical back pain). cLBP patients have abnormal FC in PAG centered pain modulation network during rest
(10) Deogaonkar M, et al. 2016	RS-fMRI performed with stimulator off and stimulator at optimum pain relief settings; Seed based analysis of FC for elements in pain network and DMN	10 CPRS or neuropathic leg pain	Same group	Decreased connection strength between somatosensory and limbic areas and increased connection strength between somatosensory and DMN with optimal SCS resulting in pain relief.	SCS reduced the affective component of pain resulting in optimal pain relief. Suggests that pain relief from SCS may be reducing negative emotional processing associated with pain, allowing somatosensory areas to become more integrated into default mode activity Heterogeneous population, no blinding, no true control group for comparison
(12) Hemington KS, et al. 2016	RS FC MRI Cross-network connectivity analysis between the DMN and SN	20 AS (3 F, 17 M)	20 (3 F, 17 M)	Patients exhibited less anticorrelated FC between SN and DMN, and the degree of cross-network abnormality tracked pain and disease-related symptoms. Sensorimotor cortex cross-network FC correlated with measures of physical function.	Suggests that cross-network FC may be a metric of functional brain abnormality in chronic pain. Physical functioning also impacts brain network interaction in chronic pain.
(13) Loggia ML, et al. 2013	ASL fMRI Independent Component Analysis to investigate RS connectivity on ASL data	16 CLBP (11 F, 5 M)	16 (11 F, 5 M)	cLBP patients demonstrated stronger baseline DMN connectivity to the pregenual anterior cingulate cortex (pgACC), a component of the MPFC, as well as to the left inferior parietal lobule. The strength of DMN-pgACC connectivity within this cluster was negatively correlated with clinical pain at baseline (r = -0.73, p = 0.001) There was a stronger DMN-insula connectivity in the cLBP patients	The performance of calibrated physical maneuvers induced changes in pain, which were paralleled by changes in DMN-INS connectivity. Greater clinical pain at baseline was associated with greater DMN connectivity with the insula and less connectivity with the pgACC.
ROI = region of interest SBP = subacute back pain M = males F = females NAc = nucleus accumbens INS = insula PFC = Prefrontal cortex VBM = voxel-based morphometry rsFC = resting state functional connectivity		DMN = default mode network SN = salience network AS = ankylosing spondylitis PAG = periacqueductal gray FC = Functional connectivity Pre-Cu = precuneus ACC = anterior cingulate cortex LP = lateral parietal region SMG = supramarginal gyrus		CRPS = complex regional pain syndrome OA = osteoarthritis ASL = Arterial Spin Labeling ICA = Independent component analysis pgACC = pregenual ACC vmPFC = ventromedial PFC vlPAG = ventrolateral PAG	

Since previous fcMRI studies have been based on heterogeneous populations of non-specific cLBP patients with different inciting pain events and unknown pain phenotypes (neuropathic vs. nociceptive or constant vs. intermittent pain), our second goal was also to study a more homogeneous group of cLBP patients with a common inciting event of previous back surgery. Moreover, we studied a specific subpopulation of these failed back surgery syndrome (FBBS) patients with refractory, constant, neuropathic cLBP. These patients are considered as clinically ‘end-stage” to the extent that they have extensively navigated through the healthcare system and have exhausted and failed all other treatment modalities to the point that they are being considered as potential candidates for spinal cord stimulation (SCS). Patients who are referred to our comprehensive multi-disciplinary chronic pain center undergo extensive evaluations and are provided with behavioral therapies and treatments prior to consideration for spinal cord stimulation. SCS is a safe, effective treatment that administers doses of electrical current to the spinal cord for the management of refractory chronic pain with over 30,000 patients receiving SCS for treatment of chronic pain each year.[13] SCS can provide satisfactory long-term pain relief for these patients in up to 60–70% of cases. [14, 15] However, in standard clinical practice, prior to implantation of a permanent SCS therapeutic system, patients are required to show significant pain improvement during a simple 3–7 day temporary test period of stimulation. We specifically studied these patients that had significant pain reduction during this required test period or “SCS trial”, yet prior to undergoing implantation of a permanent SCS system and thus prior to any ongoing SCS therapy.

We hypothesize that these FBSS patients with end-stage constant, neuropathic cLBP that are being considered for SCS therapy have altered FC between different RSNs, specifically those involved in emotion and reward/aversion functions. We also hypothesize these changes in FC are related to pain levels. The group of patients studied here represents the most clinically homogeneous population of chronic pain patients ever studied with functional imaging.

## Materials and methods

### Institutional approval

The Medical College of Wisconsin/Froedtert Hospital Institutional Review Board approved this study. Written informed consent was obtained for all participants of the study.

### Inclusion and exclusion criteria

Non-pregnant, English-speaking adult FBSS patients with constant, severe, neuropathic cLBP who passed a trial of SCS were included in the study. Patients who were non-English speaking, pregnant, or otherwise unable to undergo MRI due to safety reasons or claustrophobia were excluded from the study.

### Study protocol

Adult patients with a diagnosis of failed back surgery syndrome (FBSS) who had undergone a spinal cord stimulation (SCS) trial with positive effects (i.e. reported improvement in their pain control during the trial) and therefore thought to be good candidates for long-term SCS were screened for our study. The patients who met the inclusion criteria and wished to participate in our study signed an informed consent and were asked to fill out a questionnaire prior to completing their imaging study where they were asked to rate their level of pain using the visual analog scale (VAS) from 0–10, where 0 meant no pain and 10 meant the worst pain imaginable.

Anatomical and RS fcMRI scans were completed on all FBSS patients prior to their planned surgery for implantation of a permanent SCS system. Anatomical and RS fcMRI for healthy age-matched control subjects were obtained from the healthy control subject MRI data bank from a previous study.[16]

### Imaging methodology and data analysis (Fig 1)

**Fig 1:**
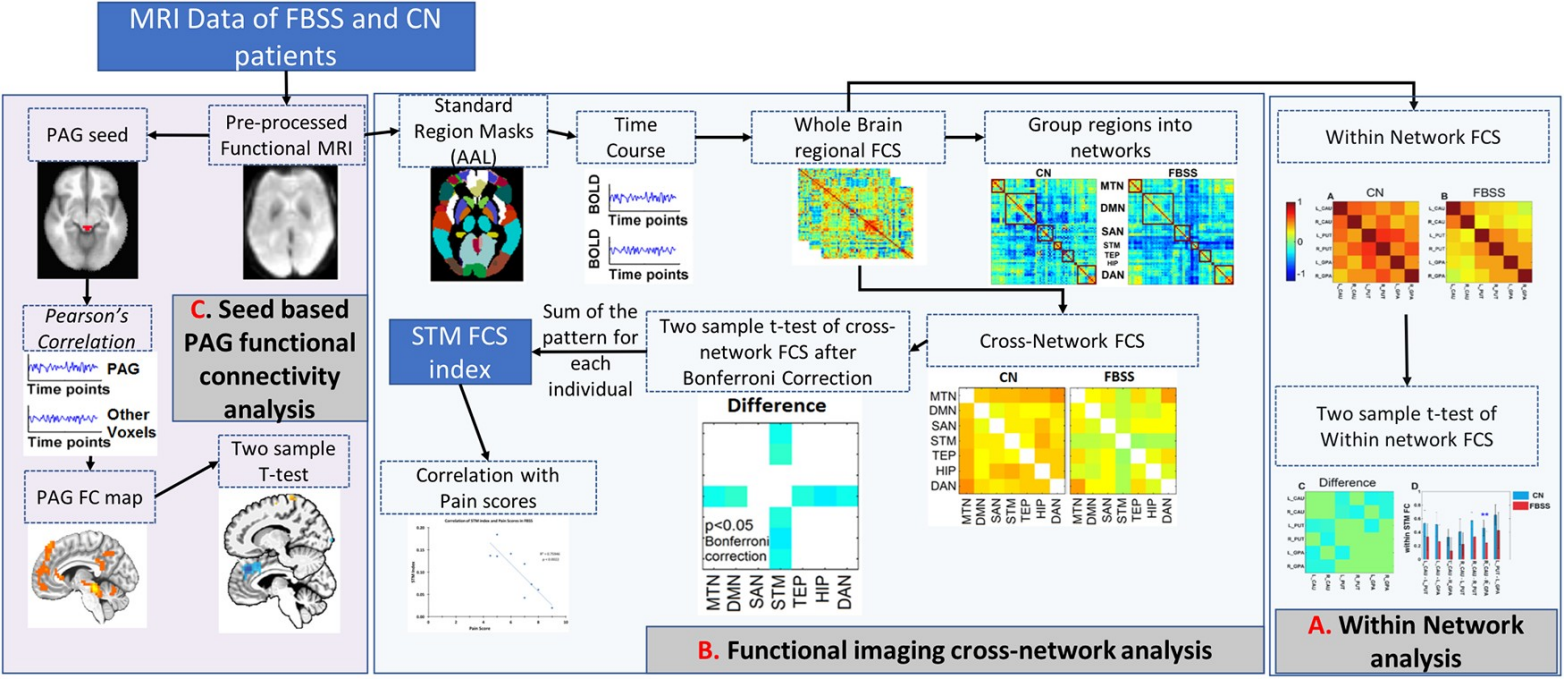
Summary of methodology and data analysis. **A** Regional FC within STM network for CN, FBSS. A two-sample t test is performed to analyze the difference in regional FC between CN and FBSS. **B**. Functional imaging cross-network analysis. Start from the pre-processed functional MRI imaging, using SPM to register the imaging into standard template with anatomical automatic labeling (AAL) maps. Each brain region’s BOLD signal is extracted and correlated with each other region’s BOLD signal to construct the whole brain regional FC network. The regions are then grouped into seven well known networks, i.e. MTN, DMN, SAN, STM, TEP, HIP and DAN. The FC across the seven networks (cross-network FC) is calculated for each individual. Two-sample Wilson-rank test is performed to characterize the difference between FBSS and control (CN) group. The *STM*_*FCS*_ index is calculated individually based on the difference pattern and then correlated with the pain level scores. **C**. Seed-based PAG FC analysis. Each subject’s PAG whole brain FC is obtained by first extracting PAG ROI using MNI coordinates.[19, 20] This is followed by ROI co-registration to the functional data. The time series of all voxels within the co-registered region of interest (ROI) are averaged to obtain each subject’s PAG time series. Voxelwise Pearson cross-correlation coefficients (CC) between the seed region and the whole brain are calculated and then subjected to a Fisher transformation to improve normality. Finally, a two-sample student t-test is used to compare PAG FC between the CN and FBSS groups and results are corrected using the AlphaSim program.

#### Image acquisition

The imaging studies were acquired using a Discovery MR750 3T Signa GE scanner with a standard quadrature transmit receive head coil. During the resting state acquisitions, the study participants were asked to close their eyes and relax. Modest acquisition parameters were selected to enable comparison with previously acquired data, and to allow for the potential of protocol-matched imaging of these participants following the placement of SCS without RF irradiation of the implanted device (data not included in this study). Sagittal RS fcMRI datasets of the whole brain were obtained in 6 minutes with a single-shot gradient echo-planar imaging (EPI) pulse sequence. The fcMRI imaging parameters were: TE/TR/flip angle/slices: 25ms, 2s, 90° and 36. The slice thickness was 4mm. Matrix size is 64×64 with 24cm field of view. Anatomical reference used the High-resolution 3D-SPGR axial images which has 144 slices, slice thickness 1mm, and 256×256 resolution. The respiration and heart beat signals were recorded using a respiratory bellows and pulse oximeter for the data analysis in the RETROICOR nuisance regression as implemented in AFNI (3dRETROICOR), to reduce signal variance associated with physiologic noise.[17, 18]

#### Resting state functional imaging data preprocessing

The data processing were performed by the software of Analysis of Functional NeuroImages (AFNI) (http://afni.nimh.nih.gov/afni/) and MATLAB (Mathworks). First, the first five volumes of the raw functional imaging data were removed for T1 equilibration. Second, corrected the time shift caused by the Interleaved slice acquisition-dependent using the AFNI command, *to3d*). The third, the AFNI command (*3dDespike)* were used to remove the Spikes in time series data. The fourth step is the motion correction. The fifth step is to remove the trend signal AFNI commands: *3dDetrend*).

The whole brain was segmented into 116 anatomical regions using the Atlas template ^25^. This resulted in 116 mapped ROIs. The average time course within each ROI was extracted from the RS functional imaging datasets. The white matter and CSF signals were extracted based on the standard masks (http://afni.nimh.nih.gov/pub/dist/data/TT_wm+tlrc) and (http://afni.nimh.nih.gov/pub/dist/data/TT_csf+tlrc) and together with whole brain global mean signals were regressed out from the 116 regional average time series.

#### Definition of networks

Seven RSNs were analyzed: motor network (MTN), default mode network (DMN), salience network (SAN), striatum network (STM), temporal network (TEP), hippocampus network (HIP) and dorsal attention network (DAN). Detailed brain regions for each network are listed in Table 2. The TEP and HIP networks were separated from the memory network as we found that hippocampal regions had significant gray matter density (GMD) changes in the FBSS group compared to healthy control patients (S1 Fig, unpublished data). The relationship between these RSNs and neurodegeneration, neuropathic pain, and chronic pain have been well reported in previous studies [1, 2, 5, 6, [21–30]].

**Table 2:** The brain regions of each resting state network (RSN).

MTN	DMN	SAN	STM	TEP	HIP	DAN
L/R_precentral gyrus	L/R_superior frontal gyrus, dorsolateral	L/R_rolandic operculum	L/R_caudate nucleus	L/R_transverse temporal gyri	L/R_hippocampus	L/R_inferior occipital
L/R_supplementary motor area	L/R_superior frontal gyrus, orbital part	L/R_insula	L/R_putamen	L/R_superior temporal gyrus		L/R_fusiform gyrus
L/R_postcentral gyrus	L/R_middle frontal gyrus, lateral part	L/R_anterior cingulate gyrus	L/R_globus pallidus	L/R_superior temporal pole		L/R_superior parietal lobule
L/R_precuneus	L/R_middle frontal gyrus, orbital part	L/R_middle cingulate		L/R_middle temporal pole		L/R_inferior parietal lobule
L/R_paracentral lobule	L/R_opercular part of inferior frontal gyrus	L/R_parahippocampal gyrus		L/R_inferior temporal gyrus		L/R_supramarginal gyrus
	L/R_area triangularis	L/R_amygdala				L/R_calcarine sulcus
	L/R_orbital part of inferior frontal gyrus					L/R_cuneus
	L/R_superior frontal gyrus, medial part					L/R_lingual gyrus
	L/R_superior frontal gyrus, medial orbital part					L/R_superior occipital
	L/R_posterior cingulate gyrus					L/R_middle occipital
	L/R_angular gyrus					
	L/R_middle temporal gyrus					

#### Regional (within network) functional connectivity strength (FCS)

**Fig 2:**
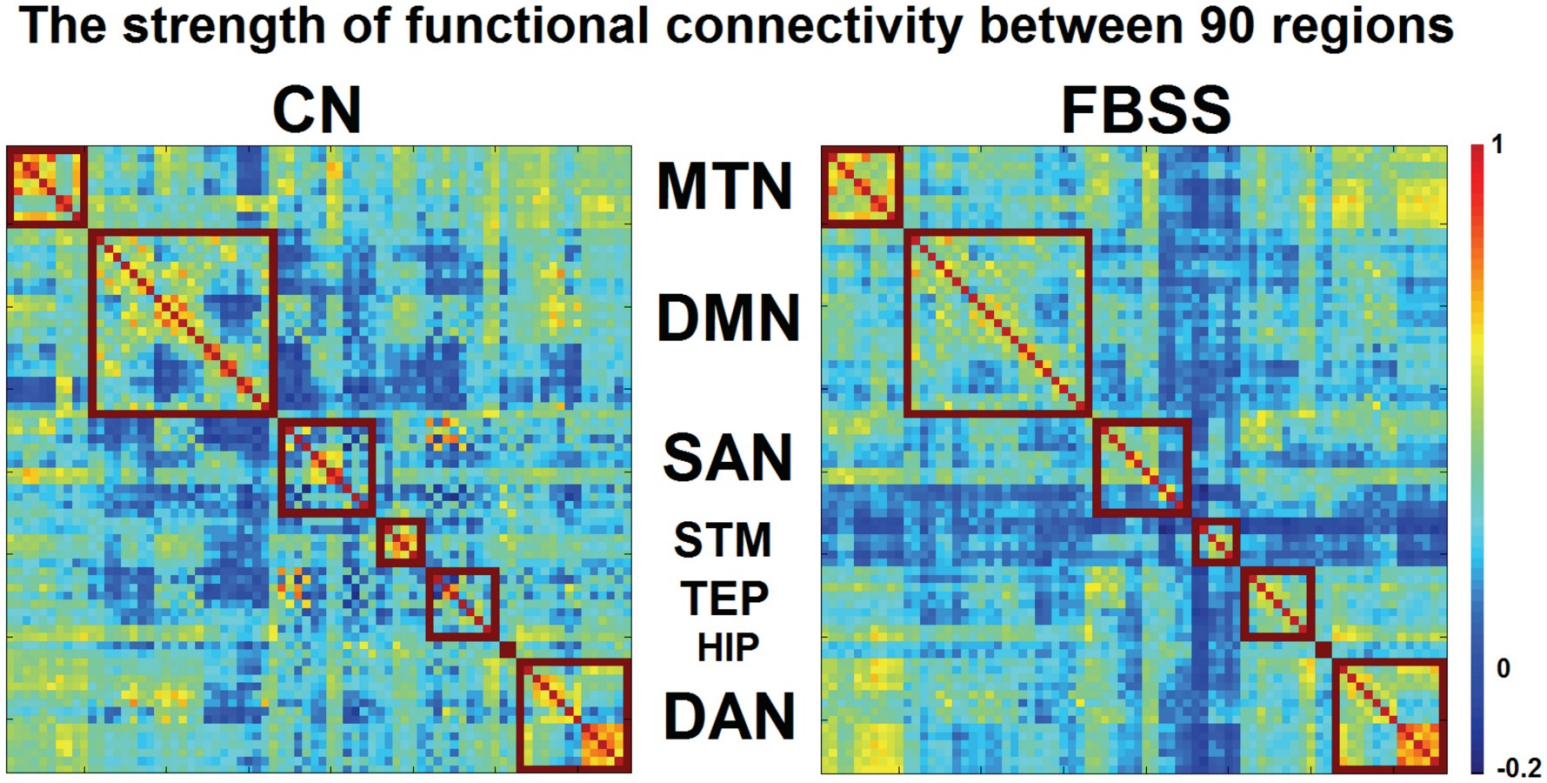
Regional-based FC between each pair of brain regions for CN group and FBSS group. A color bar (right) shows the color and associated value of FC (range from -0.2 to 1.0). Each red square contains the regions for each network.

**Fig 3:**
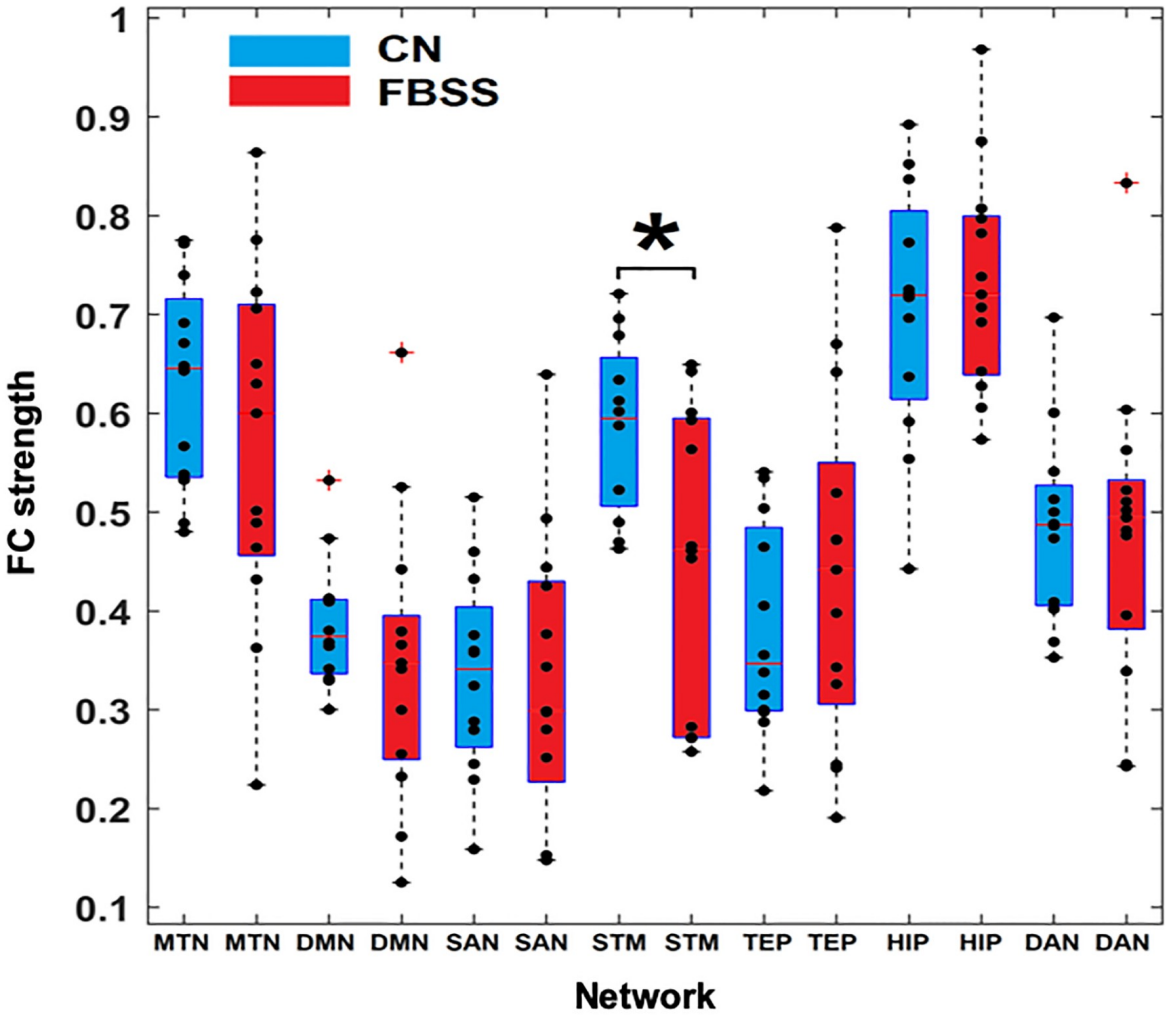
Functional connectivity (FC) within each network for control and FBSS groups. Box plots showing within network FC strength for control (**CN, blue**) and FBSS group (**red**) with individual overlaid data (**black dots**). Only the STM network shows significantly decreased within network FC (uncorrected) in FBSS group compared to CN group. * indicates p<0.05.

**Fig 4:**
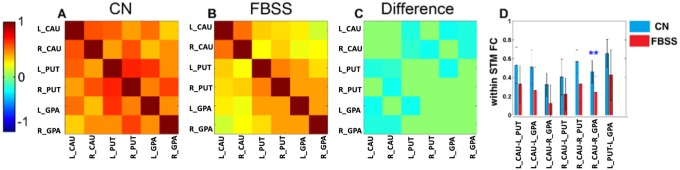
Regional FC within STM network for CN (A), FBSS (B) and the difference (C). The values of FC between left caudate and left putamen, left caudate and left globus pallidus, left caudate and right globus pallidus, right caudate and left putamen, right caudate and right putamen, right caudate and right globus pallidus, left putamen and left globus pallidus significantly decreased in FBSS group compared to CN. After using the Bonferroni method, the only significant decrease in FC between CN and FBSS groups within STM was between right caudate-right globus pallidus (** indicates p<0.05). Legend: Left and right caudate (L_CAU and R_CAU), left and right putamen (L_PUT and R_PUT), left and right globus pallidus (L_GPA and R_GPA).

The regional FCS was calculated by the Pearson correlation coefficient (CC) between the BOLD time series from two brain regions for each subject. The time series of each region were obtained by averaging the time series of all voxels within the region for each subject (Fig 2). Based on our findings (Fig 3), we further specifically tested the difference of the regional FC within STM network between CN and FBSS group (Fig 4). There are 6 regions within the STM network: left and right caudate (L_CAU and R_CAU), left and right putamen (L_PUT and R_PUT), left and right globus pallidus (L_GPA and R_GPA), so there are $\frac{n*(n-1)}{2}=\frac{6*(6-1)}{2}=15$ pairs of FC comparisons.

The Wilcoxon-rank test was performed for each pair of FC between the CN and FBSS groups. The Bonferroni method (p<0.05) was applied to evaluate significance of the difference in FCS between CN and FBSS groups.

#### Cross-network FCS

The cross-network FCS between two network A and B were produced by the mean of all possible regional FCs between Ai and Bi. Ai and Bi are brain regions that belong to network A and network B, respectively.

### Statistical analysis

#### Statistical analysis for cross-network FCS

The non-parametric Wilcoxon signed-rank test was performed to test group differences of each cross-network FCS. Multiple comparison correction was performed using the Bonferroni method with p<0.05 to avoid false positives.

#### STM functional connectivity strength (*STM*_*FCS*_) index

The *STM*_*FCS*_ index is a network-based singular index of rsFC, obtained by averaging of all cross-network FCS between STM and the other six networks. The use of a network-based singular index of rsFC as a biomarker for evaluating the functional connectivity strength of a specific disease process has been proven reliable and has been validated in various brain disease processes in our previous studies. [16, 28] The rationale of averaging different network FCS into a singular index is to reduce the noise and variation. A previous study addressed the fact that network-based analysis can enhance the signal-to-noise (SNR) and reproducibility of resting-state FC data.[29] Additionally, by using network-based functional connectivity, the number of false positive cross-correlations can be significantly reduced due to the reduced number of the total pairs of correlations.

To test if the STM_FCS_ idenx is related to pain in FBSS patients, we applied a linear regression model (*STM*_*FCS*_ *index* = *β*_0_ + *β*_1_ * *PL* + *ε*) between STM_FCS_ index and pain scores for each subject. The PL is the Pain Level easement. The *β*_0_ is the intercept of the fitting curve. The *β*_1_ is the effect of the pain level. The *ε* denotes random errors. We defined the *STM*_*FCS*_ index to have a significant linear relationship with PL if the model had p<0.05.

#### Periaqueductal grey (PAG) seed-based functional connectivity (FC) analysis

Each subject’s PAG whole brain FC was obtained as follows: First, the PAG ROI were separately extracted with the MNI coordinates of x = 1, y = −29, z = −12 provided by a previous study.[19] The ROI seed was a 4mm diameter sphere around the coordinates. The PAG seed ROI was then co-registered to the functional data. The timeseries of PAG seed were obtained by averaging the time series of all voxels within the co-registered ROI for each subject. Voxelwise Pearson cross-correlation coefficients (CC) between the seed region and the whole brain were calculated (AFNI: 3dfim+) and then subjected to Fisher transformation to improve normality [m = 0.5ln(1+CC)/(1-CC)].

#### PAG FC comparison between groups

A two-sample student t-test was used to compare PAG FC between the CN and FBSS groups (AFNI: 3dttest++). The result pattern from the two-sample t-test was corrected using the AlphaSim program (cluster size > 6000mm^3^, α = 0.01, voxelwise *p*<0.05).

## Results

### Population characteristics

A total of 26 FBSS patients who passed a SCS trial were screened. Of these, 6 patients declined the study and 10 patients were unable to undergo MRI due to safety reasons or claustrophobia and were therefore excluded from the study. Anatomical and RS fcMRI scans were performed on the remaining 10 FBSS patients (mean age 54.4yr ± 9.6yr, male:female ratio 4:6) prior to permanent implants. (Table 3). This experimental design including highly restrictive enrollment criteria yielded a well-characterized study group, while limiting opportunity for subsequent exploratory and multi-variate analysis. Anatomical and RS fcMRI for 12 healthy age-matched control subjects (mean age 56yr ± 7.2yr, male:female ratio 3:9) were obtained from the healthy control subject MRI data bank from a previous study.[16] (Table 4) Medication intake was not controlled at the time of the scans.

**Table 3:** Population characteristics for FBSS patients.

Patient ID	Age	Sex	Location/Type of pain	Pain Duration (years)	Time from latest spine surgery to SCS trial (years)	Time from SCS trial to fcMRI (days)	Time from SCS trial to permanent system implant (days)	Spontaneous Pain Level (VAS 0–10)	STM_FCS_ Index	Medications
1	51	F	Low back, groin, and bilateral legs (radicular)	10	0.5	58	73	7	0.118	N
2	47	F	Mid to low back, left hip & left knee (radicular)	20	2.3	28	31	4.5	0.137	N, NSAID
3	68	M	Low back, left buttocks & left calf (radicular)	45	1.9	33	42	7.5	0.073	N
4	68	M	Right hip and buttocks	1	2.1	17	49	5	0.135	N, MR
5	40	F	Low back, right hip, right leg (radicular)	4	0.7	45	59	9	0.019	N, MR, A
6	45	F	Low back and left leg (radicular)	1	0.7	31	35	7	0.042	NSAID, MS, MR
7	50	M	Low back and neck	35	1.2	16	21	5	*N/A	NSAID, A
8	60	F	Low back	15	4.6	28	31	8	0.060	MS, A
9	33	M	Low back, right buttocks, right leg (radicular)	2	1.9	29	52	5	0.184	N, NSAID, MR, MS
10	61	M	Low back, bilateral legs (radicular)	6	1.1	33	35	6	0.141	N, MS

Narcotic (N); Muscle relaxant (MR); Membrane Stabilizer (MS); Anxiolytic (A); Non-steroidal anti-inflammatory (NSAID)

*N/A = not available, unable to obtain due to imaging artifacts

Visual analog scale (VAS) (0 = no pain, 10 = maximum imaginable pain)

**Table 4:** Population characteristics for healthy control patients.

Patient ID	Age	Sex	STM_FCS_ Index
013	61	F	0.1125
017	65	F	0.2861
034	58	M	0.302
035	53	F	0.3628
036	47	M	0.3322
038	51	F	0.1955
039	61	F	0.2392
040	64	F	0.3009
041	46	M	0.3367
042	65	F	0.0626
045	49	F	0.4964
046	53	F	0.2933
048	63	F	0.118

### Regional-based (within network) functional connectivity strength

As opposed to the cross-network FC, within network FC strength is the average value of all possible connections between regions within each network. Only the STM network showed significantly decreased within network FC (uncorrected) in FBSS group compared to CN group (p<0.05). (Fig 3). The FCS between left caudate-left putamen, left caudate-left globus pallidus, left caudate-right globus pallidus, right caudate-left putamen, right caudate-right putamen, right caudate-right globus pallidus, and left putamen-left globus pallidus was significantly decreased in the FBSS group compared to CN (Fig 4). After using the Bonferroni method, the only significant decrease in FC within STM between the CN and FBSS groups was between right caudate-right globus pallidus.

### Decreased cross-network FCS in FBSS

**Fig 5:**
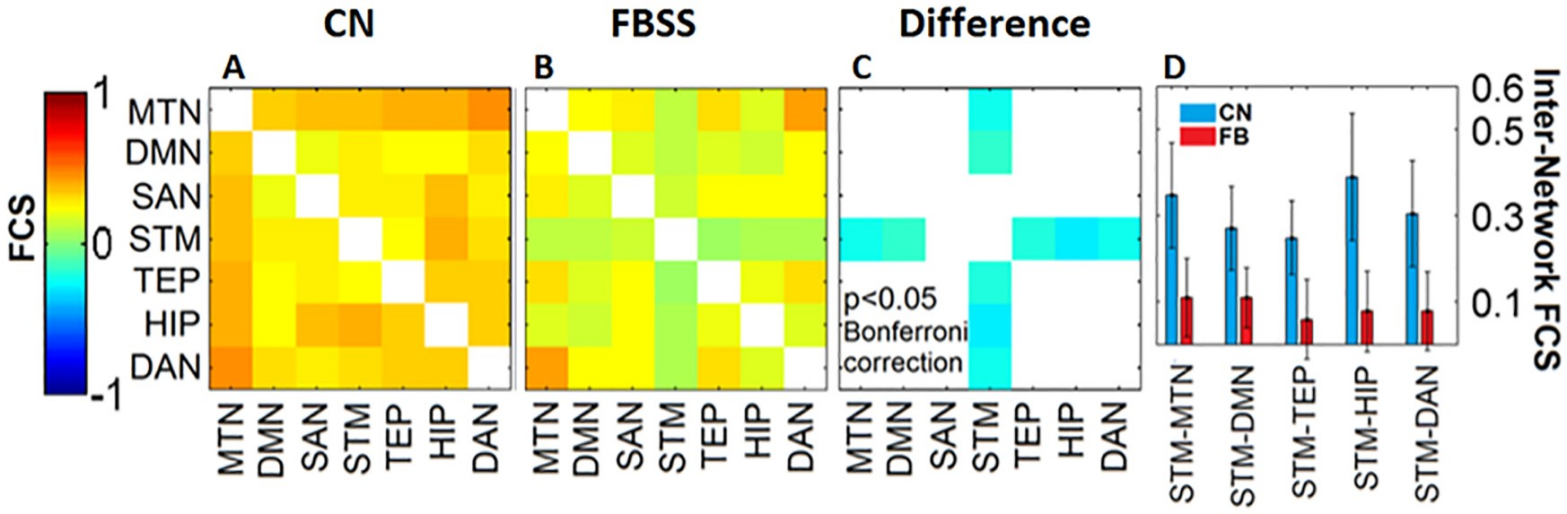
Cross-network FC among RSNs for CN group (A), FBSS group (B), and difference (C), respectively. A color bar (left) shows the color and associated value of FC. D. Bar graph with mean and standard deviation values of FC between STM and MTN, DMN, TEP, HIP and DAN for the CN group (blue) and FBSS group (red).

The FCS across all seven networks (i.e. cross-network connectivity) for the CN and FBSS groups are depicted in Fig 5. Each element within the matrix represents the cross-network FCS. The diagonal within each matrix has no value (network self-connection). The warm and cold colors represent the positive and negative FCS, respectively. Matrix analysis showed stronger positive FCS among the CN group. A Wilcoxon signed-ranked test for each pair of connections between the CN and FBSS groups (Fig 5C) revealed significantly decreased FCS (p<0.05 Bonferroni corrected) between the STM-MTN, STM-DMN, STM-TEP, STM-HIP, and STM-DAN in the FBSS group.

#### Correlation of STM-FCS with pain scores

**Fig 6:**
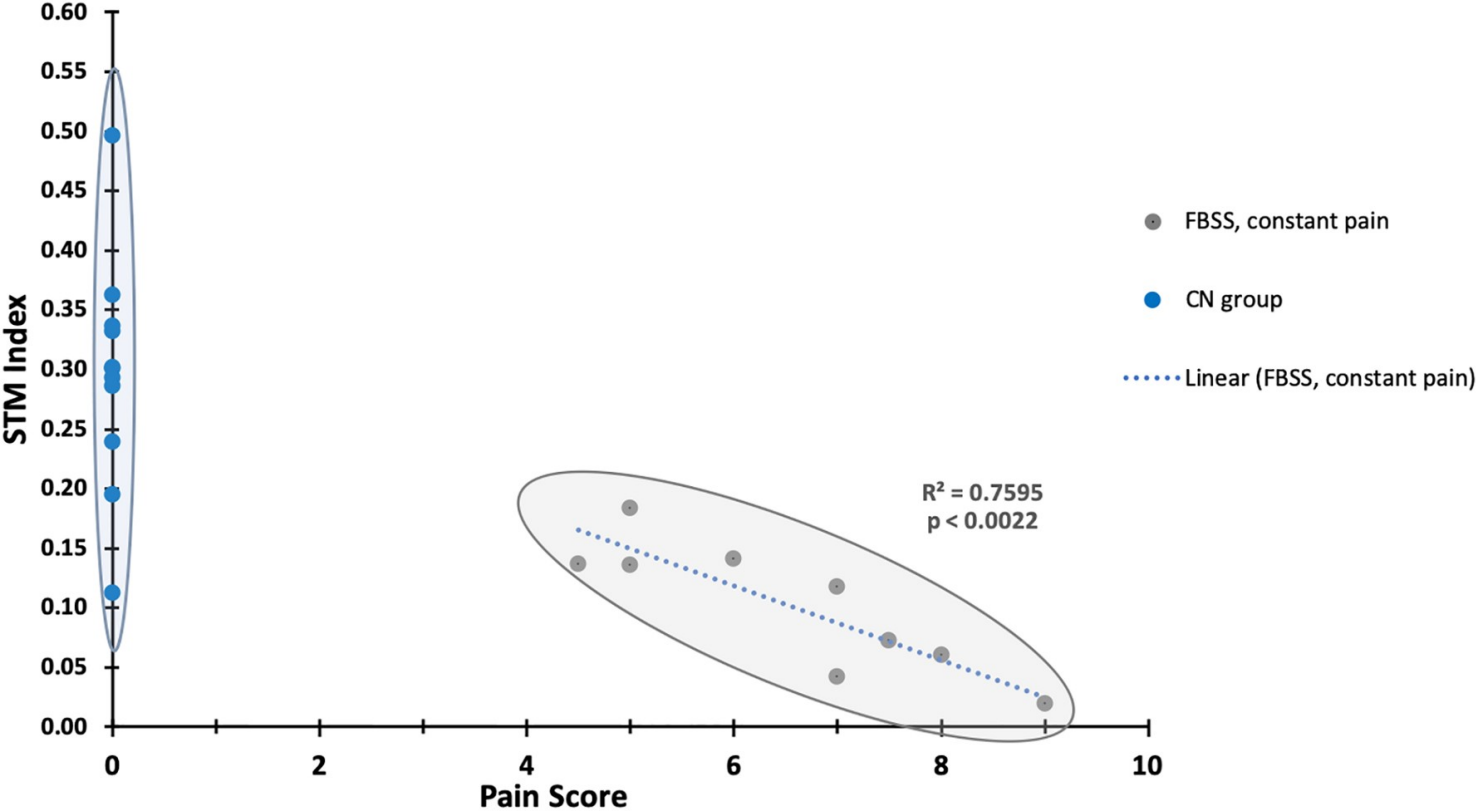
Clinical significance of *STM*_*FCS*_. A negative linear relationship was found between *STM*_*FCS*_ and the corresponding pain scores in FBSS group (**gray**). *STM*_*FCS*_ index is the mean FC between STM and all other 6 networks. Mean *STM*_*FCS*_ index for control (**CN, blue**) group is 0.27, STD 0.13.

To seek if the *STM*_*FCS*_ index was related to pain in our patients, we applied a linear regression model between *STM*_*FCS*_ index and the pain score for each subject. A significantly negative linear relationship (R^2^ = 0.76, p<0.0022) was found between *STM*_*FCS*_ index and the corresponding pain scores in FBSS subjects, indicating that smaller STM_FCS_ indices associates with higher pain scores (Table 2, Fig 6).

#### Altered FC of PAG in FBSS

**Fig 7:**
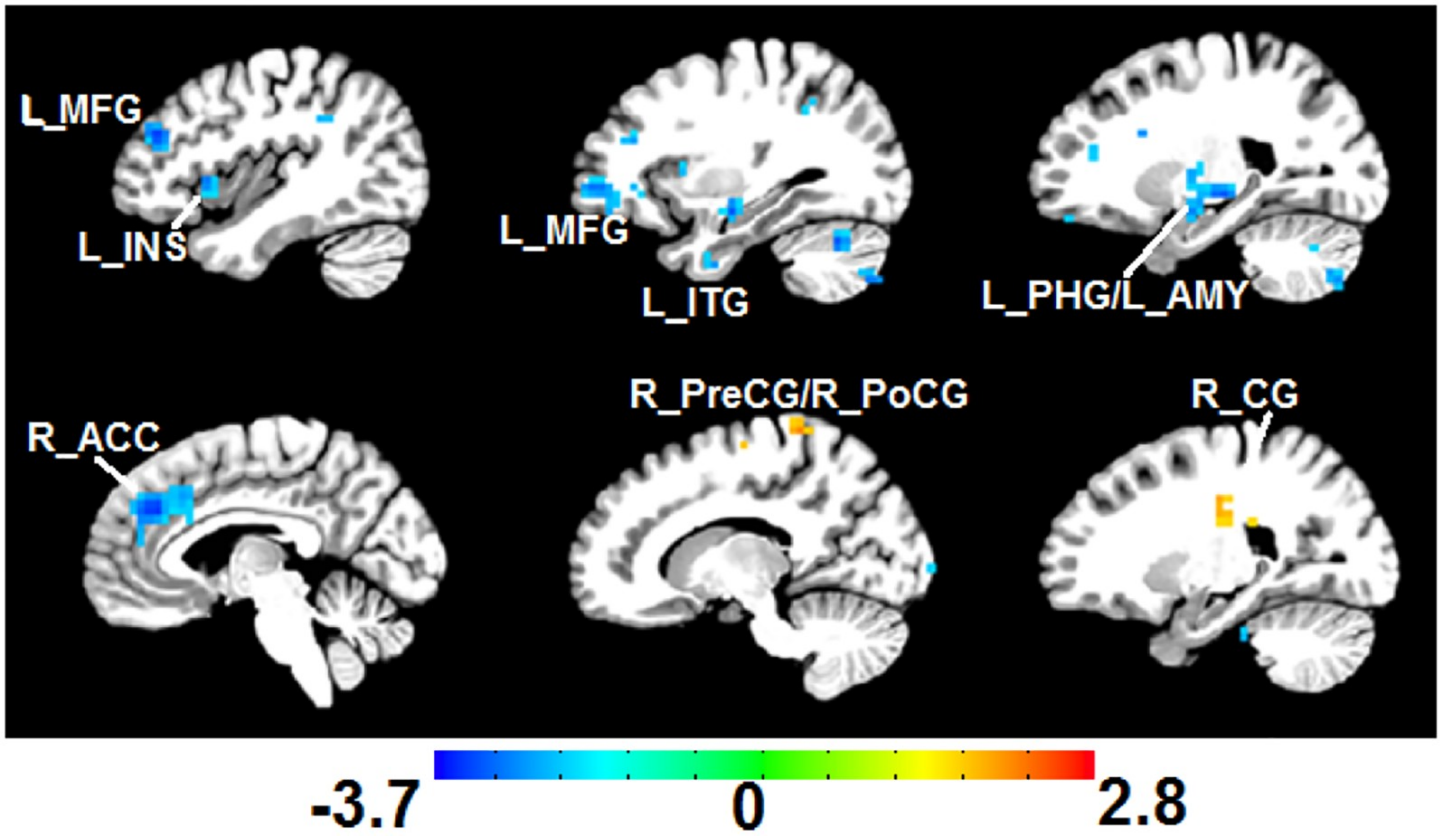
Altered PAG FC in FBSS. PAG FC was significantly decreased (cold color) in L_MFG, L_INS, L_ITG, L_PHG, L_AMY, and R_ACC. FC was increased (warm color) between PAG and the R_PreCG, R_PoCG, and R_CG when compared with the CN group (*p*<0.05, AlphaSim correction). The color bar represents the corresponding Z scores. Legend: Periaqueductal gray (PAG), Left middle frontal gyrus (L_MFG), left insula (L_INS), left inferior temporal gyrus (L_ITG), left parahippocampal gyrus (L_PHG), left amygdala (L_AMY), right anterior cingulate cortex (R_ACC), right precentral gyrus (R_PreCG), right post central gyrus (R_PoCG), right cingulate gyrus (R_CG).

Compared with the CN group, the FBSS group showed altered FC between the PAG and several other regions (Fig 7).

## Discussion

### Chronic pain

The societal costs of chronic pain are overwhelming, yet its science has remained rudimentary and the success rate for its treatment is poor.[30] Its prevalence has increased worldwide to affect more than 15% of the world population and 30% of the US population.[31] Opioids remain the mainstay of treatment, despite very limited long-term efficacy, leading to abuse, addiction and death and the opioid epidemic remains a very pressing health crisis. Non-specific chronic low back pain (cLBP) is one of the most common reasons for physician visits in the USA, is a leading contributor to job-related disability and missed work [32], is characterized by a lack of recognizable pathology, and is especially difficult to treat.[7] Chronic pain research has been hampered due to the lack of an objective diagnostic test or biomarker that can complement its subjective assessment. Resting state functional connectivity MRI (fcMRI) may serve as a tool for evaluating chronic pain states. This study offers a step toward building a body of preliminary results in characterizing such an objective diagnostic test.

### Functional connectivity

We observed decreased FC between the STM network and other networks, suggesting that the most significant alteration of cross-network connectivity primarily involves a network associated with emotion/motivation/reward functions. Cross-network connectivity alterations in pain patients has been reported in one previous study, where a decrease in FC anti-correlation was seen between the SAN and DMN networks in ankylosing spondylitis (AS) patients, however the STM network was not assessed.[12] We did not observe this in our group of patients, suggesting that alterations in cross-network FC may be dependent upon the pain phenotype.[1] Our other findings (decreased connectivity between the medial prefrontal cortex (MPFC; element of DMN) and the PAG (an element of the descending inhibitory, anti-nociceptive pain system; Fig 7) are consistent with previous intra-network studies [1, 3], and may represent a unifying framework in which chronic pain involves a shift from more somatosensory pain matrix elements to more emotion/motivation/reward pain connectome elements.[11] The reorganization of the DMN seems specific to each type of chronic pain phenotype, reflecting different emotional, attentional, and cognitive abnormalities [1]. We further speculate that changes may be dependent upon pain phenotypes (e.g. it may be affected by memory of the inciting event of pain, what proportion of the pain is neuropathic vs. nociceptive, constant vs. intermittent etc.).

Some of the previously reported DMN FC changes occurred only after a pain duration of 10–15 years, suggesting that a putative, “end-stage” chronic FC pattern may be associated with the suffering of refractory pain for a prolonged period of time (see Fig 5 of Baliki et al., 2014.[1]) We observed decreased FC between the PAG and elements of the DMN and SAN, suggesting that in our end-stage group of chronic pain patients, there may be only residual or subdued influences upon this pain-modulating center. Similar decreased FC between PAG and insula and amygdala has been reported before but mainly in patients with cLBP pain duration of > 5 years [7] or in migraineurs with a history of allodynia and other neuropathic pain qualities[19, 20]. Thus, a more progressed, “end-stage” form of chronic pain with clearer neuropathic qualities may be associated with these connectivity changes and that after long-term cLBP suffering, the body is adapted to the situation and thus the modulation mechanisms are somehow weakened.[7] Continued living with chronic pain can distort the interplay amongst multiple brain networks.[1]

We also observed an increase in FC between the PAG and SS cortex (especially near the cortical representation of the back), in our group of cLBP patients as compared to controls, consistent with an altered descending inhibitory pain system especially as it pertains to back representation in the SS cortex. Although this has not been reported before for cLBP, previous studies have demonstrated altered cortical representation of painful CRPS limbs and other chronic pain states that can be normalized with effective treatment such as mind-body interventions, physical therapy, or therapeutic SCS.[33–36]

In this work, hypothesis driven analysis is presented based upon *a priori* defined regions of interest. Given the limited sample size, this analysis is preferable for this data. As more data are acquired in larger studies, data driven analysis, like independent component analysis, will offer an opportunity to further evaluate this hypothesis and, potentially, inform alternative or supporting hypotheses.

### Objective pain measures

Correlations between altered FCS and severity of pain have been reported (Table 1). Recently, a close relationship between cross-network connectivity of DMN and SAN and pain intensity in patients with AS was reported (Table 1)[12, 37]. We observed a strong negative correlation when using cross-network analysis involving the STM and other combined networks (Fig 6). Such an analysis has not been reported before. The correlation reported here may have been stronger because 1) cross-network analysis was performed and 2) a more homogenous population with a common pain phenotype was studied. We hypothesize that a spectrum of brain imaging-based quantitative objective measures of pain may exist, each specific to a particular pain phenotype.

### STM FCS, reward/aversion balance, and pain threshold

Apkarian and colleagues have elegantly demonstrated how brain activity reorganizes with transition from acute, to subacute, early and late cLBP states, including a shift in activity from brain regions involved in acute pain to more emotion or reward circuitry.[1–3, 5] As described above, some brain activity reorganization occurs only after several years of cLBP.[1, 7] Consistent with this concept, we speculate that as the patients’ cLBP remain chronically refractory, they enter into a clinically “end-stage” refractory chronic pain state (ESRCPS), similar to the group of patients studied in the present report.

**Fig 8:**
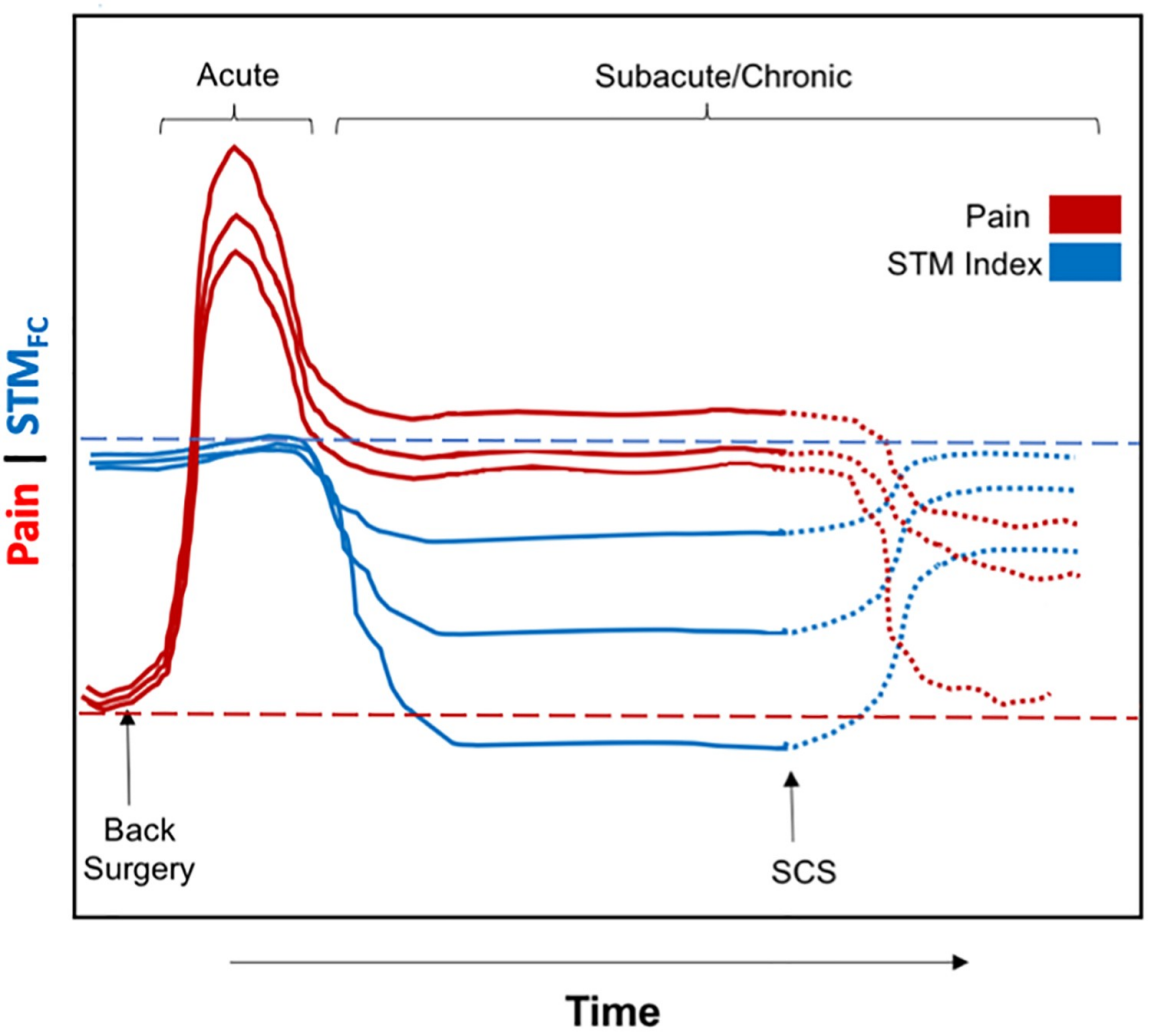
Conceptualization of changes in *STM*_*FCS*_ and pain levels seen in ESCRPS FBSS patients. In our study patients, back surgery represents the common inciting event that acutely increases pain levels (**solid red**) from baseline (**dashed red**). Subsequently, some patients (FBSS) go on to develop persistent pain (subacute/chronic phase) and enter the ESCRPS with sustained pain amplification (**solid red**) and associated decreased ***STM***_***FCS***_ index (**solid blue**) from normal baseline (**dashed blue**). Improvement in pain levels (**dotted red**) as well as normalization of ***STM***_***FCS***_ index (**dotted blue**) may be seen with successful SCS therapies.

Baliki and Apkarian (2015) have recently proposed that a chronic pain threshold (**θ**) is envisioned to be generated through connections between the MPFC, hippocampus and striatum, modulated by limbic and cortical inputs.[2] The threshold phenomenon emerges from the counterbalance between reward and aversion within the context of previous and contemporary pain experiences that then translates subconscious to conscious pain. Those with corticolimbic risk factors will develop a lower threshold and persistent pain amplification (see Fig 4 of Baliki and Apkarian (2015)). We hypothesize that in our ESRCPS patients, the STM_FCS_ index may represent a quantitative measure of the proposed **θ** value.[2] Those with a lower STM_FCS_ index will have a lower threshold for persistent pain (Fig 8). We envision this concept as being most applicable to patients with constant, neuropathic pain phenotypes (such as in patients studied in here) as opposed to intermittent non-neuropathic pain (such as mechanical back pain) or other intermittent pain phenotypes such as neurogenic claudication, in which patients can experience predictable, significant pain-free episodes by controlling their physical activities. It may be possible that decreased connectivity between the amygdala and PAG and between the STN network and other networks can occur in ESRCPS patients. Aversion/reward is counterbalanced and a lowered nociception-pain threshold (**θ**) develops with increased reported pain scores. Although the patients studied here did not receive SCS, we speculate that long-term successful SCS therapy may restore the abnormal altered connectivity patterns to more normal levels such that therapeutic benefits of SCS may be associated with normalization of the STM_FCS_ index (Fig 8).

One other group has studied similar end-stage chronic pain patients with fcMRI.[10] Ten patients with implanted SCS systems were studied with their devices turned on and off. The general trend was that therapeutic stimulation decreased connectivity between SS areas and limbic/emotional networks and increased integration of SS regions into the DMN, consistent with normalization of some of the altered connectivity previously reported for back pain.[3] However, the group of 10 patients was heterogeneous, cross-network analysis was not performed, no comparisons to normal controls were made, and connectivity patterns prior to implant were not known.

### SCS and STM

SCS has been used commonly on the type of pain patients reported here; however, about a third of implanted patients lose efficacy within 1–2 years.[14, 15] The long-term benefits of SCS may be limited by suboptimal parameters of stimulation. Recent use of alternative high-frequency or bursting patterns of stimulation may be more effective than tonic 30–50 Hz stimulation and may be due in part to direct influences on the more affective, emotional aspects of pain perception.[11, 38] There may exist a spectrum of chronic pain phenotypes that can be characterized by signatures of abnormal brain connectivity patterns that may be more amenable to certain patterns of SCS (i.e. tonic, 30–50 Hz for the somatosensory-driven and phasic, bursting-type patterns for the more affective, emotional components of pain perception).[1, 5, 11] In the chronic pain state, a dysfunctional reward system alters the balance between the two ascending and one descending pain inhibitory pain pathways and burst stimulation may be more effective in restoring or normalizing these two dysfunctional systems.[11] This dysfunctional reward system (**θ**; STM_FCS_ index) might be more amenable to correction with alternative patterns of SCS. Therefore, the STM_FCS_ index may be used to help select patients for SCS with different stimulation parameters (tonic vs. bursting). Therapeutic benefits maybe associated with normalization of the STM_FCS_ index (Fig 8).

### Study limitations and future directions

We were unable to examine the effects of age, sex or smoking because of our small sample size which is due, in part, to our narrow inclusion criteria. With this in mind and to avoid false positives, our cross-network functional connectivity analysis was subjected to rigorous multiple comparison correction utilizing the Bonferroni method. The duration of pain varied greatly in our FBSS patients which may compromise the homogeneity of our study population. However, we did not see a correlation between duration of pain and changes in connectivity patterns. Furthermore, some of the variation in pain duration may be due to the time it can take for any given patient to eventually navigate towards a SCS healthcare provider. Since we studied a very specific group of patients, our results may not be generally applicable to other pain patients. We did not control for pain medications during imaging studies. However, even with patients on heterogeneous profiles of medications (Table 2), the inverse STM_FCS_ index pain level relationship continued to be tightly correlated. By studying patients prior to their SCS trial, we may be able to determine whether functional imaging may be predictive, not only of the outcome of a temporary SCS trial (perhaps to the point of supplanting the actual trial itself), but also of the long-term outcomes of SCS. Cross-network FC studies could be extended to patients with other pain phenotypes (i.e. CRPS, mechanical back pain). A profile/spectrum of imaging-based pain biomarkers may exist for a given patient with a given pain phenotype that could help guide choice of personalized treatment options (i.e. drug vs. behavioral vs. neuromodulation therapies). The progress of an intervention could be monitored with subjective clinical assessments in combination with changing imaging-based pain biomarker status. Future studies with higher temporal resolution on the order of seconds or less with the use of electrophysiological techniques or MEG may serve to complement fcMRI studies [33, 34] and may help direct the development of other alternative stimulation patterns (such as pink or white noise) whether provided with SCS, deep brain stimulation or transcranial magnetic stimulation (TMS) techniques.[39, 40] Finally, one can speculate that fcMRI/MEG studies could be used to predict prior to a pain intervention (such as drug therapy, injections or surgery) or after such an intervention, which patients might develop or progress to a chronic pain state. In this way, one could intervene (i.e. behavioral therapy, TMS or SCS) as a preventative measure prior to its full development and, for example, decrease the incidence of FBSS.

## Conclusions

We present the first report of altered cross-network functional connectivity involving emotion/reward brain circuitry that is negatively correlated with individual patient pain scores. This measurement may represent a quantitative, objective measure of chronic pain specific to FBSS patients with moderate to severe constant, neuropathic back pain. Based on previous reports, our new findings suggest that a spectrum of image-based biomarkers may exist that are associated with a range of chronic pain phenotypes based on inciting events, pain pathology, history, quality, consistency, location, and duration and patient emotional state. A profile of such biomarkers could potentially be developed and catalogued to help guide management and prevention of different chronic pain conditions.

## Supporting information

S1 Fig Gray matter density (GMD) alterations in FBSS. A: Surface rendering figure showing the GMD changes in the FBSS group. Warm and cold color depict increasing and decreasing GMD changes, respectively. B Boxplot showing GMD changes in L/R PreCG for CN and FBSS groups. C. Boxplot showing GMD changes in L/R HIP/PHG for CN and FBSS groups. Legend: Precentral gyrus (PreCG), hippocampus (HIP), parahippocampal gyrus (PHG).
(TIFF)

S1 File (DOCX)

## Abbreviations

AAL: Anatomical Automatic Labeling
ACC: anterior cingulate cortex
AFNI: analysis of functional neuroimages
AMY: amygdala
AS: ankylosing spondylitis
BOLD: blood oxygen level dependent
CAU: caudate
CBP: chronic back pain
CC: correlation coefficients
cLBP: chronic low back pain
CN: control
CNS: central nervous system
CRPS: complex regional pain syndrome
DMN: default mode network
EPI: echo-planar imaging
ESRCPS: end-stage refractory chronic pain state
F: female
FBSS: failed back surgery syndrome
FC: functional connectivity
fcMRI: functional connectivity magnetic resonance imaging
FCS: functional connectivity strength
fMRI: functional magnetic resonance imaging
GMD: gray matter density
GPA: globus pallidus
HIP: hippocampus network
INS: insula
ITG: inferior temporal gyrus
LBP: low back pain
M: male
MEG: magnetoencephalography
MFG: middle frontal gyrus
MPFC: medial prefrontal cortex
MR: muscle relaxant
MRI: magnetic resonance imaging
MTN: motor network
N: narcotic
N/A: not available
NSAIDs: non-steroidal anti-inflammatories
PAG: periaqueductal gray
PHG: parahippocampal gyrus
PL: pain level
PoCG: post central gyrus
PreCG: precentral gyrus
PUT: putamen
ROI: region of interest
RS: resting state
rsFC: resting state functional connectivity
RSN: resting state network
SAN: salience network
SCS: spinal cord stimulation
SNR: signal-to-noise ratio
SPGR: spoiled gradient
SPM: statistical parametric mapping
STM: striatum
TEP: temporal network
TMS: transcranial magnetic stimulation
USA: United States of America
VAS: visual analog scale
