# Supplementary figures and images for: Functional connectivity and structural analysis of trial spinal cord stimulation responders in failed back surgery syndrome
Source: PLoS One. 2020 Feb 19;15(2):e0228306. doi: 10.1371/journal.pone.0228306 (PMC7029839; doi:10.1371/journal.pone.0228306)

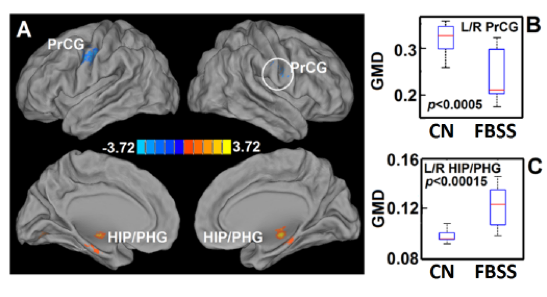

Supplement: S1 Fig — A: Surface rendering figure showing the GMD changes in the FBSS group. Warm and cold color depict increasing and decreasing GMD changes, respectively. B Boxplot showing GMD changes in L/R PreCG for CN and FBSS groups. C. Boxplot showing GMD changes in L/R HIP/PHG for CN and FBSS groups. Legend: Precentral gyrus (PreCG), hippocampus (HIP), parahippocampal gyrus (PHG). (TIFF) [file pone.0228306.s001.tiff]
