# Supplementary material for: Functional connectivity and structural analysis of trial spinal cord stimulation responders in failed back surgery syndrome
Source: PLoS One. 2020 Feb 19;15(2):e0228306. doi: 10.1371/journal.pone.0228306 (PMC7029839; doi:10.1371/journal.pone.0228306)
Supplement: S1 File — (DOCX) [file pone.0228306.s002.docx]

**Supplementary Information:**

**Structural image processing:**

Gray matter density (GMD) was calculated by Voxel-based morphometry (VBM) toolbox in Matlab with SPM8 (Wellcome Department of Cognitive Neurology, London, UK).^20^ This method can detect the differences of GMD between two groups. The VBM processing included: skull removing, segmentation of gray matter (GM), white matter (WM), and cerebrospinal fluid (CSF), special registration into Montreal Neurological Institute (MNI) space; DARTEL normalization; nonlinear modulation and smoothing using a Gaussian kernel with full-width at half-maximum (FWHM) of 8mm.

**Results:**

**Altered GMD in FBSS group:**

The FBSS group showed significantly decreased GMD in bilateral precentral gyri (PrCG) compared with the control group (CN). The brain regions with cold color in Figure S1A represent the decreased GMD regions in FBSS shown quantitatively in Figure S1B (p<0.0005). The FBSS group showed significantly increased GMD in bilateral hippocampus (HIP) and parahippocampal gyrus (PHG) regions compared to the CN group.


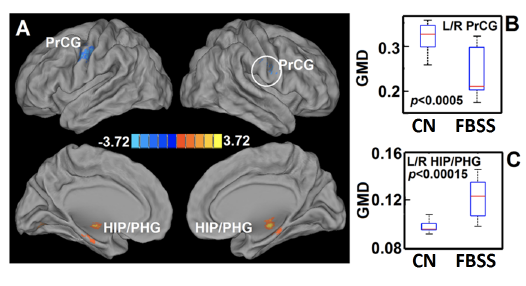


Figure S1: GMD alterations in FBSS. A: Surface rendering figure showing the GMD changes in the FBSS group. Warm and cold color depict increasing and decreasing GMD changes, respectively. B Boxplot showing GMD changes in L/R PrCG for CN and FBSS groups. C. Boxplot showing GMD changes in L/R HIP/PHG for CN and FBSS groups. Legend: Precentral gyrus (PrCG), hippocampus (HIP), parahippocampal gyrus (PHG)
